# Pediatric urology: Development, eligibility, practice

**DOI:** 10.4103/0971-9261.55150

**Published:** 2009

**Authors:** M. Bajpai

**Affiliations:** Department of Pediatric Surgery, All India Institute of Medical Sciences, New Delhi-110 029, India. E-mail: bajpai2@hotmail.com

William Ladd, Robert Gross, and Sir Dennis Browne founded the Pediatric Specialty when they realized that surgery in children demanded a dedication and a set of skills that was completely different from the adult surgical practice.

## DEVELOPMENT

In India, the concept of developing further subspecialization in this specialty was deliberated by the Heads of Departments, while preparing the Indian Association of Pediatric Surgeons (IAPS) curriculum and the syllabus for the M.Ch. training program in Pediatric Surgery, in 1998. It was unanimously felt that barring few centers in this country, there was neither the clinical workload nor sufficient infrastructure to start an efficient Fellowship Program in neonatal surgery, urology, oncology, neurosurgery, or plastic surgery.[1] It was felt that, when the need was recognized for training in a special field, trainees from the related branches could be given a chance to opt for it.

That was 10 years ago.

While efforts should continue to improve training facilities in medical colleges, training of teachers should also be enhanced.[2] It is the primary responsibility of tertiary care centers to produce teachers.

These and other goals were set in 1998 by establishing the Indian Society for Pediatric Urology (ISPU). [*http://www.pediatric-urologyonline.com*].[3] Its *motto* was to improve the training and education of surgeons in practice of children, by ‘raising the bar’. In 1998, it was considered the best way to shift gear before steering to accreditation programs.

The outcome has not been disappointing: the awareness has improved and on an average there are at least two to three meetings annually, in India, addressing all or some aspect of pediatric urology. The forum is open to those thriving to hone their skills and refurbish their knowledge, irrespective of their affiliations. Progress in Pediatric Urology is the annual education manual of ISPU and receives contributions from a galaxy of academia in Pediatric Urology, from all over the world. ISPU members include pediatric surgeons as well as adult urologists. Pediatric surgeons are excited to improve their training in a subspeciality that forms almost 40% of their practice. It is their primary training that is getting a boost. PHASE I has largely been accomplished in the first 10 years.

Similar to all fields in medicine, pediatric urology is in a constant flux and state-of-the-art care is continuously being revisited. There is an urgent need for certificate of accreditation. The idea is not to monopolize, but to give a structure to the training programs. Adult-oriented health care has established itself much earlier. With endoscopy and laparoscopy coming of age and adult-oriented instruments gaining refinements with miniaturization, these have added a shot in the arm of pediatric practice. Extracorporeal Shock Wave Lithotripsy (ESWL) and endourological techniques developed in adult practice are rapidly replacing open stone-surgery in children. Pediatric surgeons, who primarily deal with reconstructive surgery and are highly skilled in lower urinary tract endoscopy, are rapidly adding these to their armamentarium.

The process of training / certification / accreditation needs to be handled, not on whims and arrogance intending to monopolize, but with studied perseverance and a holistic approach, by weaving joint efforts, similar, if not identical to the model in Europe.[4] This would ensure the interest of the subspeciality for the benefit of development of advanced pediatric urological care.

## ELIGIBILITY

The mandate of the teaching institutions is: who would practice and how to educate and not about who should practice, as they have no regulatory control at present. The ideal setting for management of a child's problem is a children's hospital.[5]

It is globally accepted that pediatric practice has to be exclusive. Any mixing with adult practice produces unfavorable results in children.[6,7] These observations explain why pediatric urology has completely moved to pediatric surgeons in some countries and is the focus of imaginary ‘turf wars’ in others.[6–9]

There is an urgent need to bring about uniformity in training programs across the country. Sound academics is the backbone of quality output. The cutting-edge research in training programs is integral to achieving these goals. While measures for population control are, hopefully, gathering momentum by the efforts of health authorities, specialists in child care should gear themselves for the task at hand. The sheer numbers provide us a challenge and an opportunity to innovate, refine, and simplify the management of complex malformations. This can be achieved by employing state-of-the-art technology, both in research and interventions, and enable us to lead the campaign at global pedestals.

How a program is navigated depends upon the perception of goals. In response to a thought-provoking article[10] in the Journal of Indian Association of Pediatric Surgeons (IAPS), the association commissioned a symposium in Thiruvananthapuram: ‘Is Pediatric Surgery a sinking speciality?’ The stalwarts voiced their critique as custodians and dismissed the apprehensions. We must recognize that change is the only constant; shift in paradigm is inevitable, and with time, science and technology will continue to evolve in an inherent attempt to balance requirements.

In the present scenario, the number of training institutions and trained personnel are already far below the requirement.[11] The immediate plan should be the creation of units in the respective departments, exclusively for Pediatric Urology work.

If not already in existence, centers desirous of starting these programs have to ensure only one addition: the technological infrastructure for endourological procedures. The Fellows registered for training should have a recognized M.Ch. degree. If the infrastructure is still not in place they can spend one of the two years in collaboration. This approach will ensure that the subspecialist cadres are developed within the existing infrastructure, till the time the children's hospital comes into existence, in adequate numbers. When these are set up then it will be the appropriate time for starting direct M.Ch. / DNB programs in Pediatric Urology.

While Pediatric Surgery training involves at least 40% urology, adult urology training does not have any pediatric exposure. Therefore, the trainees from adult specialities who are desirous of pediatric practice will have to spend at least two years in pediatric training centers to learn the surgical management of complex malformations. Strangely, there is increasing reluctance among adultprogram trainees to join pediatric programs. Apparently, this is due to skepticism of conversion from high earning areas to ones with reduced income; at least this is the impression one gets from the US model of training; so much for ‘market’ driven training! In an informal discussion with Mr. P. G. Ransley (personal communication) he shared the view that a background of Pediatric Surgery training could have avoided this ‘holy mess’.

## PRACTICE

Circumcision, orchiopexy, and minor scrotal problems are routinely performed by surgeons / adult urologists, especially in non-academic settings. The challenge for expertise comes only from index cases, such as, posterior urethral valves, prune belly syndrome, bladder exstrophy / cloaca, proximal hypospadias, ureteric malformation / malfunction, and other complex disorders that clearly demand special skills and experience, which comes from sound training.

After attending Pediatric Urology meetings and operative workshops, delegates with adult and pediatric backgrounds alike, return home with renewed knowledge. They resume practicing the quantum of Pediatric Urology work in their regions, as much as they can. Pediatric Surgeons are trained in a fair amount of Pediatric Urology work during their training. However, due to lack of uniformity in programs across the country, their training in Pediatric Urology is often inadequate. Those who are keen on taking up subspeciality training for accreditation may choose to do so. The accreditation system will not prevent those without specialized training from performing Pediatric Urology procedures. However, in future, when a trained Pediatric Urologist settles into practice in his region, they may consider it wiser to pass complex cases to him.[12]

In India we should refrain from ‘knee-jerk’ reaction. A balance has to be struck to achieve the best of both the worlds; delivery to masses and training the trainers.

Forty-two percent of India's population is children. One-third of child health problems are surgical in nature. Almost half of the current population of Indians, who would be adults in a decade and a half or less, are not covered by any National Health Policy which can deliver. The specialists are concentrated in the urban centric environment and their density is only increasing. While many centers train surgeons who can address most of the children's surgical problems, in a traditional way, and with lower costs, the cutting edge diagnostics and techniques are only available for the urban few. Surgical care of a neonate incurs high financial inputs. Unfortunately, it is the socioeconomically backwards who are hit most, as a majority of neonates with congenital malformations come from poor families. In the US, the federal government began its efforts to provide health insurance coverage to low-income individuals in 1965, with the creation of the Medicaid program. Subsequently steps have been taken to provide health cover to all children born after 2001 in the form of, ‘Treatment of Children Deformities Act 2001’.

Health care provisions were introduced with only one accent: ‘Service’. In human memory Sushruta and Hippocrates were the first ones to realize this. If the new scales are marked with dollars or rupees do they change the dimensions? For some, if the answer is ‘yes’, so be it; let them wind their sails. The ship is still to be steered with even stronger determination.

In a vast country like India it would take a while before trained pediatric urologists fill up the requirements. Till that time the country can ill afford to term Pediatric Surgeons or adult Urologists who are without accreditation as unqualified. Even in the US, two-thirds of the pediatricians are not subspeciality trained, yet 20% spend some time in the subspeciality field.[13] However, as the issues related to litigations begin ‘breathing down the neck’ these policies would need a re-look.
